# Metastatic pheochromocytoma and paraganglioma: a retrospective multicentre analysis on prognostic and predictive factors to chemotherapy

**DOI:** 10.3332/ecancer.2023.1523

**Published:** 2023-03-20

**Authors:** Samara T Pacheco, Mauro D Donadio, Felipe Almeida, Juan M O’Connor, Valeria de Miguel, Mariano Dioca, Jose Huaman, Arinilda C Bragagnoli, Rui F Weschenfelder, Paola M Beltran, Rachel P Riechelmann

**Affiliations:** 1 AC Camargo Cancer Center, São Paulo, Brazil; 2 Hospital Italiano de Buenos Aires, Buenos Aires, Argentina; 3 Instituto Alexander Fleming, Buenos Aires, Argentina; 4 Instituto de Oncologia Ángel H. Roffo, Buenos Aires, Argentina; 5 Hospital de Amor, Barretos, Brazil; 6 Hospital Moinho de Ventos, Porto Alegre, Brazil; 7 Instituto Nacional Enfermidades Neoplasicas, Lima, Peru

**Keywords:** pheochromocytoma, paraganglioma, prognostic, predictive, survival, chemotherapy, metastasis

## Abstract

**Background:**

Prognostic and predictive markers in metastatic pheochromocytoma and paraganglioma (mPPGL) are unknown. We aimed to evaluate epidemiology of mPPGL, and prognostic factors of overall survival (OS) and predictive markers of treatment duration with first-line chemotherapy (TD1L).

**Patients and methods:**

Retrospective multicentre study of adult patients with mPPGL treated in Latin American centres between 1982 and 2021.

**Results:**

Fifty-eight patients were included: 53.4% were female, median age at diagnosis of mPPGL was 36 years and 12.1% had a family history of PPGL. The primary site was adrenal, non-adrenal infradiaphragmatic and supradiaphragmatic in 37.9%, 34.5% and 27.6%, respectively. 65.5% had a functioning tumour and 62.1% had metachronous metastases. Positive uptakes were found in 32 (55.2%) ^68^Gallium positron emission tomography (PET/CT), 27 (46.6%) 2-deoxy-2-[fluorine-18]fluoro-D-glucose PET/CT and 37 (63.8%) of ^131^Iodine-metaiodobenzylguanidine (MIBG) tests. Twenty-three (40%) patients received first-line chemotherapy, with cyclophosphamide, vincristine and dacarbazine used in 12 (52%) of patients. At a median follow-up of 62.8 months, median TD1L was 12.8 months. Either functional exams, tumour functionality, pathological characteristics or primary tumour location were significantly associated with response or survival. Yet, negative MIBG, Ki67 ≥ 10%, infradiaphragmatic location and functional tumours were associated with numerically inferior OS.

**Conclusions:**

In patients with mPPGL, prognostic and predictive factors to chemotherapy are still unknown, but negative MIBG uptake, Ki67 ≥ 10%, infradiaphragmatic location and functional tumours were numerically linked to worse OS. Our results should be further validated in larger and independent cohorts.

## Introduction

Paragangliomas and pheochromocytomas (PPGL) are neuroendocrine neoplasms that arise from paraganglia and chromaffin cells but of the adrenal medulla, respectively. PPGL are rare, with estimated incidence of about 0.6 cases per 100,000 people/year [1], and both can secrete catecholamines and neuropeptides, with resulting paroxysmal hypertension, constipation, episodic headache, sweating and pallor, tremors and palpitations [2]. To date, there are no established clinical, histopathological or biochemical features to determine metastatic behaviour. Hence, a diagnosis of malignant PPGL relies on the presence of metastases, which occurs in approximately 10%–20% of cases, with heterogeneous disease course [1, 3]. In an attempt to evaluate prognostic factors for malignant PPGL, a multiparameter score (PASS) was conceived for adrenal gland pheochromocytoma based on the evaluation of morphologic features of tumour cells. The PASS score, published in 2002, considered high risk of malignant behaviour when the score was ≥4 [4]. In 2014, a new score was developed, the Grading of Adrenal Pheochromocytoma and Paraganglioma (GAPP) scoring system, which added the immunohistochemistry (IHC) Ki67 index and the presence of catecholamine secretion to the PASS parameters. The score can range from 0 to 10, higher scores being linked to higher risk of metastases [5]. A recent study evaluated the accuracy of PASS, GAPP and a modified GAPP score, where investigators also evaluated the lack of succinate dehydrogenase complex iron sulphur subunit B gene (*SDHB*) IHC expression as prognostic factor for metastasis in 72 PPGL cases [6, 7]. Both GAPP scores and modified version were more accurate than PASS. Nevertheless, the reproducibility of these scores is variable because of interobserver variations [4].

While prognostic markers of malignant potential in PPGL have been studied, little is known about prognostic factors for survival and/or predictive markers of benefit from systemic therapies, even for the most commonly used first-line systemic treatment, the combination chemotherapy with cyclophosphamide, vincristine and dacarbazine (CVD). The effects of this combination were assessed in a systematic review and meta-analysis including 50 patients, showing a complete or partial response rate in 4% and 37%, respectively, and stable disease in 14% of patients [13]. No prognostic or predictive factors, however, were assessed.

At present, data on the efficacy of tyrosine kinase inhibitors in metastatic paraganglioma/pheochromocytoma are derived from phase II studies [14–16]. Sunitinib was evaluated in a European randomised, placebo-controlled trial phase II trial with malignant PPGL (FIRST-MAPPP trial). Median progression-free survival (PFS) was 8.9 versus 3.6 months [17]. In addition to chemotherapy and tyrosine kinase inhibitor (TKI), the use of nuclear medicine in treatment for metastatic PPGL (mPPGL) is well established. A meta-analysis was performed which showed a complete or partial tumour response in 3% and 27% of patients treated with ^131^Iodine-metaiodobenzylguanidine (MIBG), respectively. In addition, 52% of patients had stable disease; [18] but given the often indolent nature of pheochromocytoma and metastatic paraganglioma, it is unclear whether this is due to treatment effects or the natural history of the disease [19]. As an alternative to MIBG treatment, if the tumour is a somatostatin receptor positive upon imaging, peptide receptor radionuclide therapy (PRRT) or somatostatin analogues may be considered. A meta-analysis of 201 patients with mPPGL showed that PRRT achieved an objective response rate of 25% and a disease control rate of 84%. Clinical and biochemical responses were seen in 61% and 64% of the patients, respectively [20]. Beyond the first-line setting, temozolomide has been described as a potential therapeutic tool, mainly in the second line [21, 22].

Yet, some patients with mPPGL have indolent disease, with disease stabilisation for years, and some patients present aggressive and rapidly progressing tumours; while some patients have highly responsive tumours to chemotherapy or sunitinib, others present upfront refractory disease. Thus, prognostic and predictive markers in mPPGL are unclear. Our primary objective was to evaluate prognostic factors of overall survival (OS) and predictive markers of treatment duration with first-line chemotherapy for patients with mPPGL. Our secondary objective was to describe the epidemiology of mPPGL patients.

## Materials and methods

This was a retrospective, multicentre study of patients with mPPGL, defined by radiological exams. Consecutive patients from the seven following hospitals in Latin America specialised in cancer treatment were included: AC Camargo Cancer Center (São Paulo, Brazil), Hospital de Amor (Barretos, Brazil), Hospital Moinho de Ventos (Porto Alegre, Brazil), Instituto Nacional Enfermidades Neoplasicas (Lima, Peru), Hospital Italiano de Buenos Aires (Buenos Aires, Argentina), Instituto Alexander Fleming (Buenos Aires, Argentina), Instituto de Oncologia Ángel H. Roffo (Buenos Aires, Argentina). The protocol was approved by the Ethics Committee Boards of participating institutions. Patients over 16 years old diagnosed with histologically confirmed PPGL and radiologically documented synchronous or metachronous metastases were eligible. Cases seen only once, as second opinions, where follow-up information was not available, were excluded. Consecutive cases were selected from centres’ databases from January 1982 through September 2021, using the C47 or C74 coding of the International Classification of Diseases, 11th Revision.

The following data were collected from medical charts: gender, date of birth and date of initial diagnosis and of metastatic disease, country of origin, family history of PPGL, known genetic syndrome, smoking status, Eastern Cooperative Oncology Group (ECOG) performance status at the time of mPPGL, time interval from initial diagnosis until evidence of metastases in initially non-stage IV cases, metastatic sites, primary site location, presence of functioning syndrome, functioning imaging tests (^68^Gallium positron emission tomography (^68^Ga-PET/CT), 2-deoxy-2-[fluorine-18]fluoro-D-glucose PET/CT (^18^F-FDG-PET/CT), MIBG scintigraphy), Ki67 IHC expression index, type and lines of treatments, date of progressions, survival status on last follow-up and date of last follow-up.

Patients from AC Camargo Cancer Center with archived paraffin-embedded tumour tissues available had their pathology material revised and evaluated for the IHC expression of SDHB in a tissue microarray by a pathologist with expertise in the diagnosis of PPGL (FA).

Our main objective was to investigate prognostic and predictive factors to systemic chemotherapy, the most common regimen being utilised for mPPGL patients in our region. To increase internal validity, we limited our analytical sample to patients with mPPGL who received systemic chemotherapy in first-line. The primary endpoint was OS, which was calculated from the date of radiological documentation of metastatic disease to the date of death of any cause. The main secondary endpoint was treatment duration of disease control in first-line (DDC1L), defined as the time from day 1 cycle 1 (D1C1) of first-line chemotherapy to D1C1 of second-line treatment; patients who died before the receipt of second-line treatment, regardless of cause, were censored on the date of death. DDC1L was chosen as the main outcome variable to investigate predictive factors because we assume it is a proxy of PFS, and because we could not retrieve radiological exams for proper RECIST determination. Response to first-line chemotherapy, as documented in medical charts, was explored as an outcome variable, with patients being categorised into two groups: complete or partial response, and stable disease or upfront tumour progression.

Prognostic and predictive variables evaluated for association with OS and DDC1L were: timing of metastases (metachronous or synchronous), positive metastatic uptake on ^18^F-FDG-PET/CT, on ^68^Ga-PET/CT or on MIBG, tumour functionality, primary tumour location (infradiaphragmatic or supradiaphragmatic) and Ki67 cut-off values (≥3% or ≥10%). Synchronous metastases were defined when detected within 6 months of the initial diagnosis of localised PPGL. For the analysis of predictive and prognostic factors, we considered a Ki67 cut-off point of 3%, according to extrapolation of the GAPP score data, in which values above this level are encountered in moderately or poorly differentiated tumours. As an exploratory analysis, we also evaluated the prognostic and predictive effects of Ki67 cut-off of 10%, based on data from patients with well-differentiated gastro-entero-pancreatic neuroendocrine tumours treated with targeted agents [8]. The Ki67 index captured was preferably that from metastases, if both primary and metastatic lesions were available.

Descriptive statistics were used to summarise absolute and relative frequencies and medians. DDC1L and OS were estimated by Kaplan–Meier method. The reverse Kaplan–Meier method was used to estimate the follow-up time. The unadjusted log-rank test was used to compare OS and DDC1L times according to prognostic and predictive variables, respectively. To determine the association between tumour response and predictive variables, we used the chi-square or exact test of Fisher. For all analyses, two-tailed *p* value < 0.05 was deemed significant. All analyses were performed in SPSS version 24.

## Results

From 1980 to 2018, 58 patients with mPPGL were identified and included: 31 (53.4%) were female, median age at diagnosis of metastatic disease was 36 years (16–86), and seven (12.1%) patients had a family history of PPGL. The primary site was adrenal, non-adrenal infradiaphragmatic and supradiaphragmatic in 37.9%, 34.5% and 27.6% of cases, respectively. Thirty-eight (65.5%) patients had a functioning tumour and 62.1% had metachronous metastases. The great majority (84.9%) of patients had a ^68^Ga-PET/CT at some point during the course of their mPPGL. An ^18^F-FDG-PET/CT was performed by 36 (62%) and an MIBG was performed in 92.8% of the patients. Among these cases, positive uptakes were found in 32 (55.2%) ^68^Ga-PET/CT, in 27 (46.6%) ^18^F-FDG-PET/CT and in 37 (63.8%) of MIBG tests. Table 1 summarises the characteristics of patients.

Twenty-three (40%) patients received first-line chemotherapy and comprise the population for the evaluation of prognostic and predictive factors. Other directed therapies administered in first-line were upfront metastasectomy in 12 (21%) patients, therapeutic MIBG in 9 (15%), Lutetium^177^ in 5 (9%), a somatostatin analogue in 4 (7 %) and sunitinib in 2 (3%) cases.

### Prognostic and predictive factors in first-line chemotherapy in mPPGL

Table 2 depicts the characteristics of 23 patients treated with first-line chemotherapy. The median age was 43 years (16–71). The most commonly used chemotherapy regimen was CVD in nearly half (12; 52%) of patients. Of 21 patients with radiological response assessment, 4 (19%) achieved a partial response, 10 (47.6%) had stable disease and 7 (33.3%) had disease progression as the best response. Either tumour uptake on MIBG (*p* = 1.0), ^68^Ga-PET/CT (*p* = 1.0) or ^18^F-FDG-PET/CT (0.58), the Ki67 index (cut off 3% (*p* = 1) or 10% (*p* = 0.63)), tumour functionality (*p* = 0.63), timing of metastases (*p* = 0.63) or primary tumour location (*p* = 0.54) influenced the response to first-line chemotherapy (Table 3).

**Figure 1.**
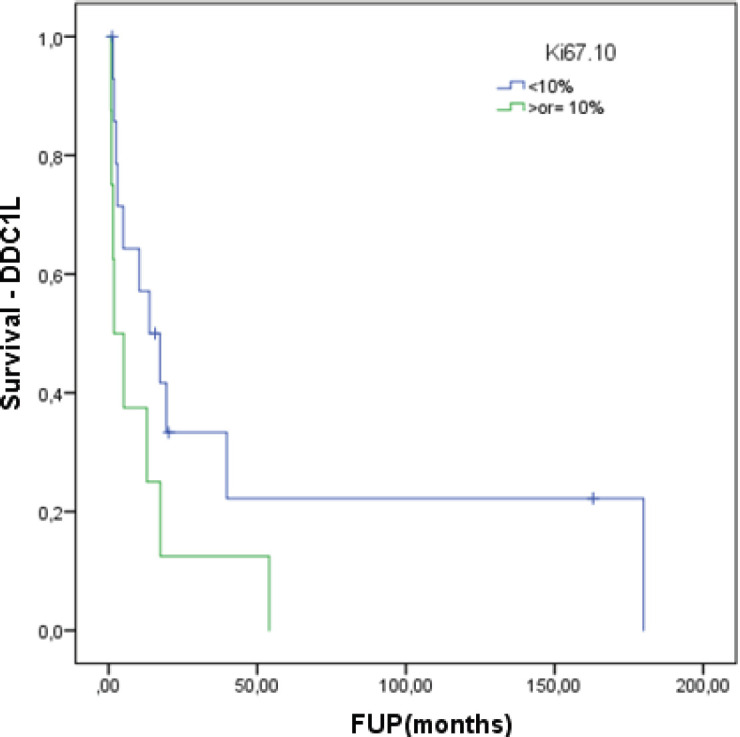
Time of treatment in first-line chemotherapy according to Ki67.

**Figure 2.**
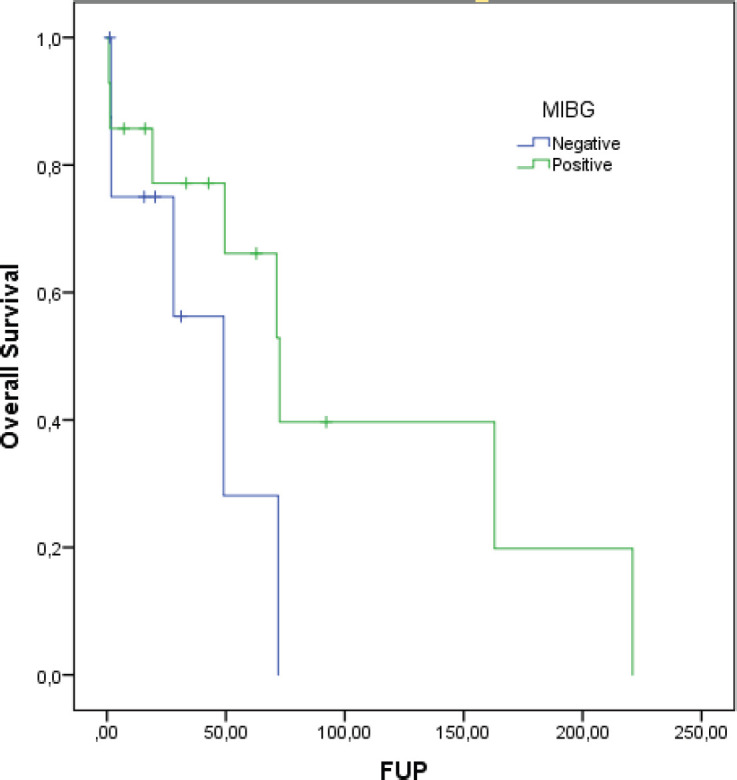
OS according to MIBG uptake.

**Figure 3.**
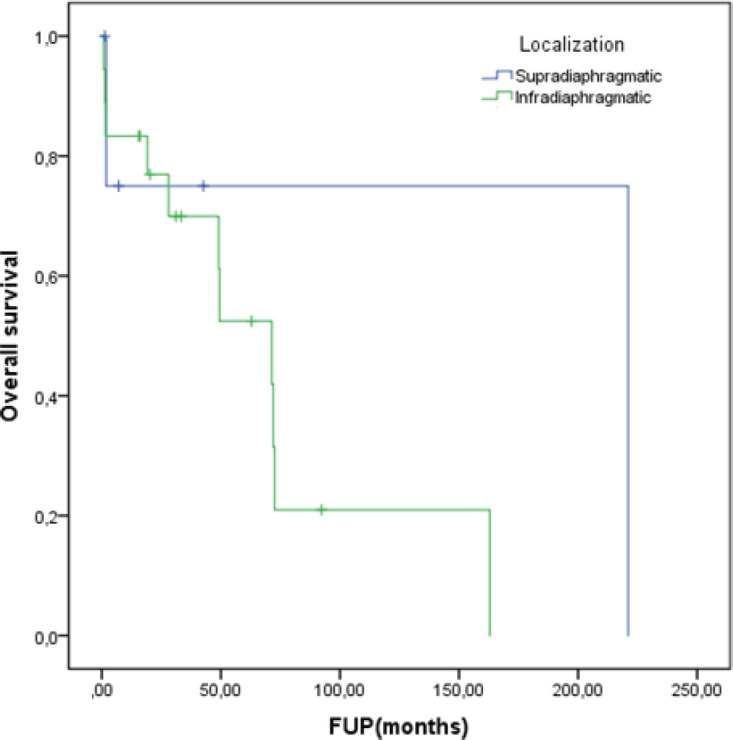
OS according to localisation.

**Figure 4.**
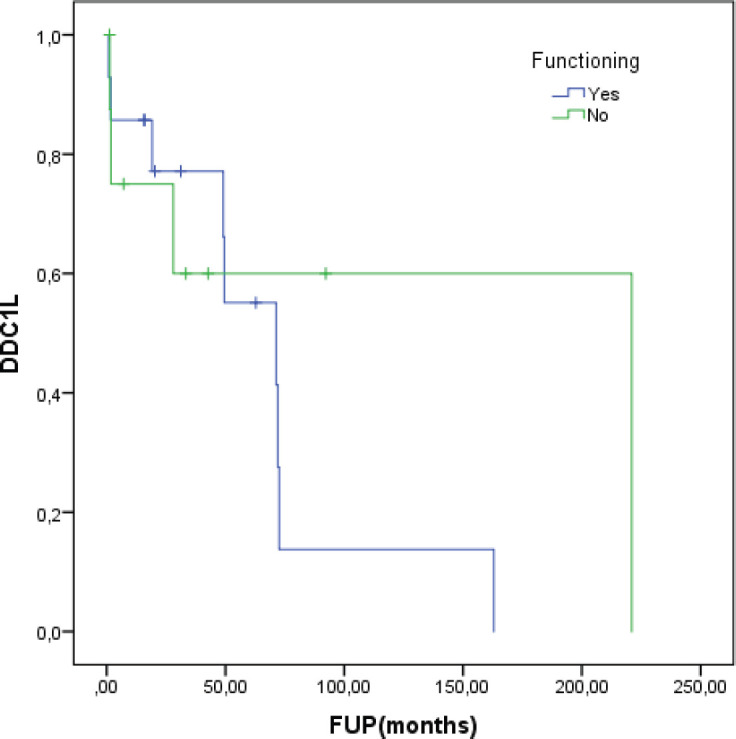
Disease control in first-line (DDC1L) according to functioning tumour.

At a median follow-up of 62.8 months, the median DDC1L was 12.8 months. During follow-up, 13 patients passed away and the median OS of all 23 patients was 71.3 months. No variable was significantly prognostic for OS or predictive of DDC1L. Non-significant large numerical differences were observed in DDC1L according to Ki67 (Ki67 < 10%: median of 13.7 months versus ≥10%: 1.8 months; *p* = 0.1; Figure 1) and OS according to MIBG uptake (median of 72.6 for positive uptake versus 42.1 months for negative uptake; *p* = 0.13; Figure 2) by primary tumour location (supradiaphragmatic location: median of 221 × 71.3 months for adrenal and infradiaphragmatic; *p* = 0.28; Figure 3); or to tumour functionality (non-functioning tumours: median of 221 × 71.3 months; *p* = 0.39; Figure 4).

## Discussion

While prognostic and predictive factors in mPPGL remain unknown, our data suggest that negative baseline MIBG uptake, Ki67 ≥ 10%, infradiaphragmatic location and functional tumours may be associated with inferior OS. Our multicentre study also reports similar epidemiology to other series of mPPGL.

Unfortunately, financial issues do not yet allow molecular and genetic analyses to be performed routinely for all patients with PPGL in developing countries. Less than 40% of our sample had *SDHB* mutation test, but the prevalence found of 24% is also consistent with studies that showed that SDH mutations prevalence estimated to lie between 10% and 30% of PPGL [9]. Only 12%, however, had a family history, which is described in between a quarter and one third of PPGL cases [7, 10, 11]. Although it may be associated with the difficulty of accessing high-quality records on family history, a possible cause for the lower frequency of family association in our country is the high miscegenation characteristic of Latin American countries, which can decrease the homozygosity of autosomal recessive syndromes, more associated with PPGL [12].

Regarding functionality, about 60% of patients treated with chemotherapy had a functioning tumour, with positivity for MIBG and low Ki67 (<10%). Despite that, the main treatment used was chemotherapy and the regimens most used were CVD (52%) and cisplatin plus etoposide (22%). While CVD chemotherapy historically provides a partial response in 37% of patients, we found 19% of response and this lower rate may be associated with the predominance of low-grade tumours in our sample. Fifteen of the 23 patients treated with first-line chemotherapy had Ki67 less than 10%.

We could not identify, however, significant factors associated with DDC1L or response to first-line chemotherapy or prognostic factors of OS among these patients. Nevertheless, we observed a trend for lower DDC1L among patients whose tumours had a Ki67 ≥ 10%, and worse OS in patients with negative tumour uptake in MIBG, infradiaphragmatic/adrenal location or with functioning tumours. These findings point to a more aggressive behaviour in these subgroups and a larger number of patients could overcome a potentially underpowered analysis of our small sample.

Because of the rarity of mPPGL, very few studies have been undertaken to investigate factors associated with treatment benefits. Most of the subsequent studies concerning Ki67 in adrenal and/or extra-adrenal pheochromocytomas were aimed at demonstrating that an elevated proliferative activity was more indicative of malignant behaviour [23–27]. In localised PPGL, the best prognostic cut-off varies in studies: some had identified 3% [23], some authors 6% [25], others 5% [26, 27]. In the metastatic setting, although Ki67 does not have a well-defined role as a prognostic marker, in our study, the data suggest, as in neuroendocrine neoplasms of the gastro-entero-pancreatic tract, a worse survival in those with Ki67 > 10%.

In an early study, most pheochromocytomas (21 out of 29 patients) accumulated 18F-FDG-PET/CT [28]. FDG uptake was observed in a greater proportion of malignant than benign tumours, and in those tumours that did not accumulate MIBG, took up FDG. The discrepancy between MIBG and FDG uptake has since been observed in other studies especially in patients with metastatic disease [29, 30]. This was postulated to be due to tumour dedifferentiation and loss of specific cellular characteristics, such as cell membrane norepinephrine and vesicular monoamine transporter systems responsible for MIBG uptake [30]. Although not significant, our study suggests that patients with positive MIBG have a longer survival, inferring a higher level of cell differentiation and less aggressive disease.

Tumour functionality may also be associated with prognosis. Some mPPGL are associated with high morbidity and mortality secondary to hypersecretion of catecholamines and metanephrines leading to hypertension, cardiovascular disease and even death [8] and, in our study, we actually found data that suggest lower OS in functioning tumours. Similarly, the European retrospective MAPP-Prono Study evaluated 169 patients from 18 centres and found that better survival was associated with head and neck paraganglioma (almost never functional), age < 40 years, metanephrines less than fivefold the upper limits of the normal range and low proliferative index. In multivariate analysis, hypersecretion (HR: 3.02 (1.65–5.55); *p* = 0.0004) was identified as an independent significant prognostic factor of worse OS [31]. SDHB mutations were evaluated but it could not be confirmed as a major prognostic parameter in PPGL and suggest additional key molecular events involved in tumour progression.

Our study has several limitations that should be acknowledged. Being a retrospective study on a rare disease, we covered a period of 39 years, where heterogeneity of certain therapeutic managements has occurred. Although we planned to enroll all consecutive cases, selection bias is possible and we do not have the exact number of excluded cases based on potential missing files. Additionally, it was not possible to collect reliable information on treatment-related adverse events, dose intensity, biochemical response or details on radiological response. Furthermore, the retrospective design did not permit us to obtain complete information about Ki67 evaluation of all centres. The small number of the enrolled patients represents another limitation, which may prevent unveiling possible predictors and to perform adjusted analyses to identify independent predictors of DDC1L and OS, although the rarity of chromaffin tumours should be considered.

Future research in rare cancer populations is possible only through collaboration. Our study is an example of such an effort conducted by members of Latin Americans institutions. Global collaborative studies are pivotal steps for the oncology community to learn the biology, patient characteristics and treatment outcomes of rare cancers.

## Conclusion

In patients with mPPGL, prognostic and predictive factors to first-line chemotherapy are still unknown. Non-significant findings suggest that negative MIBG uptake, Ki67 ≥ 10%, infradiaphragmatic location and functional tumours are associated with lower OS. Our results should be further validated in independent and larger cohorts.

## Conflicts of interest

No conflicts of interest to declare.

## Funding

This work received no funding.

## Figures and Tables

**Table 1.** Baseline characteristics – general population (all = 58).

Characteristic	*N* (%)	Characteristic	*N* (%)
Country Brazil Argentina Peru	28 (48.2) 25 (43.1) 5 (8.7)	^68^Ga-PET/CT Positive uptake Negative uptake Unknown	32 (55.2) 13 (22.4) 13 (22.4)
Age	36 (16–86)	^18^F-FDG-PET/CT Positive uptake Negative uptake Unknown	27 (46.6) 9 (15.5) 22 (37.9)
Sex Male Female	27 (46.6) 31 (53.4)	MIBG Positive uptake Negative uptake Unknown	37 (63.8) 15 (25.9) 6 (10.3)
Smoking Yes No	8 (13.8) 49 (84.5)	Metastasis Synchronic Metachronic	22 (37.9) 36 (62.1)
ECOG 0–1 ≥2	45 (77.6) 13 (22.5)	Metastasis site Liver Bone	28 (48.3) 37 (63.8)
Family history of PPGL Yes No	7 (12.1) 51 (87.8)	*SDHB* germline testing Mutated Wild type Unknown	14 (24.1) 8 (13.7) 36 (62)
Primary site Adrenal Supradiaphragmatic extra-adrenal Infradiaphragmatic extra-adrenal	22 (37.9) 16 (27.6) 20 (34.5)	Ki67 <3 3–9 ≥10 Unknown	9 (15.5) 21 (36.3) 11 (18.9) 17 (29.3)
Functioning tumour Yes No	38 (65.5) 20 (34.5)	Treatment Chemotherapy Upfront metastasectomy MIBG Lutetium^177^ Somatostatin analogue Sunitinib Not reported	23 (40) 12 (21) 9 (15) 5 (9) 4 (7) 2 (3) 3 (5)

Data are *n* (%) or median (range)

ECOG PS, Eastern Cooperative Oncology Group performance status; PPGL, Pheochromocytoma/paraganglioma; MIBG, ¹³¹Iodine-metaiodobenzylguanidine scintigraphy; SDHB, Succinate dehydrogenase complex iron sulphur subunit B

**Table 2.** Baseline characteristics – patients treated with first-line chemotherapy (*N* = 23).

Characteristic	*N* (%)	Characteristic	*N* (%)
Sex Male Female	11 (47.8) 12 (52.2)	^18^F-FDG-PET/CT Positive uptake Negative uptake Unknown	11 (47.8) 9 (39.2) 3 (13)
Smoking Yes No	2 (8.7) 21 (91.3)	MIBG Positive uptake Negative uptake Unknown	14 (60.9) 7 (30.4) 2 (8.7)
ECOG 0–1 ≥2	20 (87) 3 (13)	Metastasis Synchronic Metachronic	9 (39.1) 14 (60.9)
Family history of PPGL Yes No	2 (8.6) 21 (91.4)	Metastasis site Liver Bone	13 (56.5) 18 (78.3)
Primary site Adrenal Supradiaphragmatic extra-adrenal Infradiaphragmatic extra-adrenal	6 (26.2) 12 (52.1) 5 (21.7)	*SDHB* germline testing Mutated Wild type Not performed	5 (21.8) 8 (34.8) 10 (43.4)
Functioning tumour Yes No	14 (60.9) 9 (39.1)	Ki67 <3 3–9 ≥10	3 (13) 12 (52.2) 8 (34.8)
^68^Ga-PET/CT Positive uptake Negative uptake Unknown	7 (30.5) 13 (56.5) 3 (13)	Chemotherapy CVD Cisplatin/Etoposide Capecitabine/Temozolomide Temozolomide Cisplatin/Paclitaxel Folfox Cyclophosphamide/Adriamycin/Cisplatin	12 (52) 5 (22) 2 (9) 1 (4) 1 (4) 1 (4) 1 (4)

Data are *n* (%) or median (range)

ECOG PS, Eastern Cooperative Oncology Group performance status; PPGL, Pheochromocytoma/paraganglioma; MIBG, ¹³¹Iodine-metaiodobenzylguanidine scintigraphy; SDHB, Succinate dehydrogenase complex iron sulphur subunit B; CVD, Cyclophosphamide, vincristine and dacarbazine

**Table 3.** Predictive factors to first-line chemotherapy.

Characteristic	Response: *N* (%)	No response: *N* (%)	*p* value
Ki67 <3% ≥3% <10% ≥10%	0 (0) 4 (22) 3 (23) 1 (12.5)	3 (100) 14 (78) 10 (77) 7 (87.5)	1 0.63
MIBG Positive Negative	2 (15.4) 1 (16.6)	11 (84.6) 5 (83.4)	1
^68^Ga-PET/CT Positive Negative	1 (16.7) 2 (16.6)	5 (83.3) 10 (83.3)	1
^18^F-FDG-PET/CT Positive Negative	3 (27.2) 1 (12.5)	8 (72.8) 7 (87.5)	0.58
Functioning Yes No	3 (23) 1 (12.5)	10 (77) 7 (87.5)	0.63
Metastasis Synchronic Metachronic	1 (12.5) 3 (23)	7 (87.5) 10 (77)	0.63
Primary site Supradiaphragmatic Infradiaphragmatic	0 (0) 4 (23.5)	4 (100) 13 (76.5)	0.54

MIBG, ¹³¹Iodine-metaiodobenzylguanidine scintigraphy; all comparisons by Fisher exact test
